# Functional study of the novel multidrug resistance gene HA117 and its comparison to multidrug resistance gene 1

**DOI:** 10.1186/1756-9966-29-98

**Published:** 2010-07-19

**Authors:** Lihua Zhao, Xianqing Jin, Youhua Xu, Yuxia Guo, Rui Liang, Zhenhua Guo, Tingfu Chen, Yanhui Sun, Xionghui Ding

**Affiliations:** 1 Laboratory of Oncology, Affiliated Children's Hospital, Chongqing Medical University, No.136, Zhongshan 2nd Road, Yuzhong District, Chongqing 86 400014, China; 2 Department of Surgery, Affiliated Children's Hospital, Chongqing Medical University, No.136, Zhongshan 2nd Road, Yuzhong District, Chongqing 86 400014, China; 3 Department of Hematology, Affiliated Children's Hospital, Chongqing Medical University, No.136, Zhongshan 2nd Road, Yuzhong District, Chongqing 86 400014, China

## Abstract

**Background:**

The novel gene HA117 is a multidrug resistance (MDR) gene expressed by all-trans retinoic acid-resistant HL-60 cells. In the present study, we compared the multidrug resistance of the HA117 with that of the classical multidrug resistance gene 1 (MDR1) in breast cancer cell line 4T1.

**Methods:**

Transduction of the breast cancer cell line 4T1 with adenoviral vectors encoding the HA117 gene and the green fluorescence protein gene (GFP) (Ad-GFP-HA117), the MDR1 and GFP (Ad-GFP-MDR1) or GFP (Ad-GFP) was respectively carried out. The transduction efficiency and the multiplicity of infection (MOI) were detected by fluorescence microscope and flow cytometry. The transcription of HA117 gene and MDR1 gene were detected by reverse transcription polymerase chain reaction (RT-PCR). Western blotting analysis was used to detect the expression of P-glycoprotein (P-gp) but the expression of HA117 could not be analyzed as it is a novel gene and its antibody has not yet been synthesized. The drug-excretion activity of HA117 and MDR1 were determined by daunorubicin (DNR) efflux assay. The drug sensitivities of 4T1/HA117 and 4T1/MDR1 to chemotherapeutic agents were detected by Methyl-Thiazolyl-Tetrazolium (MTT) assay.

**Results:**

The transducted efficiency of Ad-GFP-HA117 and Ad-GFP-MDR1 were 75%-80% when MOI was equal to 50. The transduction of Ad-GFP-HA117 and Ad-GFP-MDR1 could increase the expression of HA117 and MDR1. The drug resistance index to Adriamycin (ADM), vincristine (VCR), paclitaxel (Taxol) and bleomycin (BLM) increased to19.8050, 9.0663, 9.7245, 3.5650 respectively for 4T1/HA117 and 24.2236, 11.0480, 11.3741, 0.9630 respectively for 4T1/MDR1 as compared to the control cells. There were no significant differences in drug sensitivity between 4T1/HA117 and 4T1/MDR1 for the P-gp substrates (ADM, VCR and Taxol) (P < 0.05), while the difference between them for P-gp non-substrate (BLM) was statistically significant (P < 0.05). DNR efflux assay confirmed that the multidrug resistance mechanism of HA117 might not be similar to that of MDR1.

**Conclusions:**

These results confirm that HA117 is a strong MDR gene in both HL-60 and 4T1 cells. Furthermore, our results indicate that the MDR mechanism of the HA117 gene may not be similar to that of MDR1.

## Introduction

Multidrug resistance (MDR) is a major cause of treatment failure and mortality in cancer patients. Breast cancer is the most prevalent cancer among women and the second leading cause of death in cancer. The most widely used treatment of breast cancer is chemotherapy, while the success of chemotherapy in breast cancer patients is also seriously limited by the development of MDR [1]. One well-known mechanism of MDR is the over-expression of ATP-binding cassette transporters such as multidrug resistance gene 1 (MDR1), multidrug resistance-associated protein 1 (MRP1), lung resistance protein (LRP) and the breast cancer resistance protein (BCRP) [2-7]. P-glycoprotein (P-gp), which is encoded by the MDR1, is the most extensively studied drug transporter. It is an integral membrane glycoprotein with a molecular mass of 170 kDa and has been postulated to function as a pump that removes hydrophobic anticancer agents from drug-resistant cells, thus promoting MDR [8]. The novel gene HA117 (Gene Bank accession number: AY230154), which was screened and cloned from the ATRA-resistant acute myeloid leukemia cell line HL-60/ATRA using differential hybridization and gene chip assays [9], was shown to promote MDR in the chronic myelogenous myeloid leukemia cell line K562 [10]. However, the strength and mechanism of the MDR of HA117 have not yet been elucidated, especially in solid tumor cells. Our aim in the current study was to compare the MDR strength of HA117 to that of MDR1 and to examine the possible MDR mechanism(s) of HA117 in breast cancer cell line 4T1 which is a classical representation of solid tumor cell line.

Our results suggest that HA117 is a strong MDR gene and that its MDR index is similar to that of MDR1 for P-gp substrate drugs and much higher than that of MDR1 for P-gp non-substrate drugs. In addition, using the breast cancer cell line, we show that the MDR mechanism of HA117 may not be similar to that of MDR1. As such, further studies need to be conducted to determine the mechanism of HA117 to promote MDR.

## Materials and methods

### Cell culture

The HEK 293 cell line was a generous gift from professor Tong-Chuan He (Laboratory of Molecular Oncology, University of Chicago, USA). The breast cancer cell line 4T1 was bought (ATCC, USA) and preserved in our laboratory. The cells were maintained in Dulbecco's Modified Eagle Medium/Nutrient Mixture F-12 (DMEM/F12) (Gibco, USA) supplemented with 10% fetal bovine serum (FBS, Gibco, USA) and RPMI-1640 medium (Gibco, USA) supplemented with 10% FBS (Gibco, USA), respectively at 37°C in a humidified atmosphere of 5% CO_2_. The cells were passaged approximately once every 3 days.

### Preparation of high titer adenovirus vector supernatant

Recombinant adenoviral vectors expressing green fluorescence protein (GFP) and HA117 (Ad-GFP-HA117), GFP and MDR1 (Ad-GFP-MDR1) or only GFP (Ad-GFP) were previously constructed in our laboratory [10]. HEK 293 cells were transducted with Ad-GFP-HA117, Ad-GFP-MDR1 or Ad-GFP viral supernatant at a multiplicity of infection (MOI) of 2-5. When all the cells exhibited a round morphology and approximately 80% of them were detached from the culture flask (usually 4 to 5 d post-transduction), the cells were harvested and combined. The cells were then frozen using a dry ice/methanol bath, immediately thawed in a 37°C water bath, and vortexed. A total of 4 freeze/thaw/vortex cycles were performed. After expanding for 3 cycles and purifying using density gradient centrifugation, the high titer recombinant adenoviruses Ad-GFP-HA117, Ad-GFP-MDR1 and Ad-GFP were harvested, filtered in a aseptic conditions through a 0.45-μm filter and stored at -80°C [11].

### Transduction of 4T1 cells with adenoviral vector supernatant

Logarithmic phase 4T1 cells were divided into 4 groups. Cells in group 1 were transducted with Ad-GFP-HA117 and cells in group 2 were transducted with Ad-GFP-MDR1 and served as the experimental groups. the stable transductants of these cells in the two groups are referred to as 4T1/HA117 and 4T1/MDR1. A third group of cells was transducted with empty Ad-GFP and served as a control group. the stable transductants of these cells are referred to as 4T1/GFP. Untransducted cells served as a blank control and are referred to as 4T1. The cells were plated on 96-well plates at a density of 2.0 × 10^5 ^cells/well and incubated for 16 h. The cells in experiment groups and control group were devided into 6 subgroups and transducted with adenoviral vector according to the following MOI: MOI = 1, 10, 50, 100, 500 or 1000 for each subgroup. Each subgroup contained 6 repeated pores. The transduction efficiency was quantified using fluorescence microscopy and flow cytometry 48 h after transduction.

### Detection of HA117 and MDR1 mRNA expression by reverse transcription-polymerase chain reaction (RT-PCR)

48 h after transduction, total RNA was extracted from cells in the experiment and control groups using the Tripure isolation reagent (TianGen Biotechnology, China) according to the manufacturer's instructions. RT was performed using the TaKaRa RT kit (TaKaRa Biotechnology, China). The expression levels of mRNA were normalized to glyceraldehyde phosphate dehydrogenase (GAPDH). The relative expression levels of the target genes were determined by calculating the fluorescence intensity ratio between the target gene and GAPDH. The primers used for PCR were designed according to the information from the human genomic data base and were synthesized by Invitrogen Biotechnology Company (USA). The sequences of the primers used for the amplification of HA117, MDR1 and GAPDH were as follows: HA117- (forward) 5'-CAGAGTCAGGGACTTCAGCCTTAT-3', (reverse) 5'-CTGTTTCCTTCTCACTCCCAACCA-3'; MDR1- (forward) 5'-GCTGGTTTGA TGTGCACGATGTTGG-3', (reverse) 5'-ATTTTGTCACCAATTCCTTCATTAA-3'; GAPDH- (forward) 5'-ACCACAGTCCATGCCATCACT-3', (reverse) 5'-TCCACCACCCTGTTGCTG TA-3. The PCR conditions were as follows: denaturation at 94°C for 5 min and 33 cycles of denaturation at 94°C for 30 s, annealing at 60°C for 30 s and extension at 72°C for 1 min, and a final extension at 72°C for 5 minutes. The PCR products were resolved on 2% agarose gels, and the gels were photographed. Densitometric analysis was performed using the UVP gel image analysis system (Bio-Rad, USA).

### Detection of P-gp expression using a western blotting

The cells were harvested and lysed in lysis buffer (0.5% Nonidet P-40, 10 mM Tris-HCl pH 7.4, 150 mM NaCl, 1 mM EDTA, and 1 mM Na_3_VO_4_) supplemented with protease inhibitors and 1 mM phenylmethylsulfonyl fluoride (PMSF). Approximately 100 μg of total cellular lysate was then subjected to standard sodium dodecyl sulfate polyacrylamide gel electrophoresis (SDS-PAGE). For western blotting analysis, the proteins were transferred to polyvinylidene fluoride (PVDF) membranes (Millipore Inc., USA), which were then blocked for 1 h with 8% non-fat milk in 10 mM Tris-HCl pH 7.5, 100 mM NaCl, and 0.1% (w/v) Tween 20. The membranes were first incubated with antibodies against β-actin or P-gp (both from Santa Cruz, USA) overnight at 4°C, followed by 1 h incubation with horseradish peroxidase-conjugated secondary antibody. The protein signals were detected using an enhanced chemiluminescence kit (BiYunTian Biotechnology, China) and analyzed using the Bio-Rad (USA) imaging system and associated software according to the manufacturer's instructions.

### Drug elimination experiments

The activities of HA117 and MDR1 were determined using the daunorubicin (DNR) efflux assay. For this purpose, 2.0 × 10^6 ^cells/ml from each group were incubated at 37°C in an atmosphere of 5% CO_2 _for 30 min in RPMI-1640 supplemented with 10% fetal calf serum (FCS) containing 7.5 g/ml DNR (Sigma). After two washes, the cells were transferred into daunomycin-free medium and allowed to efflux for 10 min. Then 10 μg/ml of verapamil, a P-gp inhibitor, were added to the cells to stop efflux, and the cells were washed two times. The cells were then analyzed by flow cytometry using a FACScan flow cytometer (Becton Dickinson, San Jose, CA) at an excitation wavelength of 488 nm and using 530/30 nm (green fluorescence) bandpass filters.

### Analysis of drug sensitivity using Methyl-Thiazolyl-Tetrazolium (MTT) assay assays

To assess multidrug chemosensitivity, cells in the experiment and control groups were plated on 96-well plates at a density of 3.0 × 10^5 ^cells/well and incubated for 24 h at 37°C. After this time, the medium was removed, replaced with fresh medium containing adriamycin (ADM; Pharmacia Italia S.p.A, Italy), vincristine (VCR; Wanle Pharmaceutical Factory, China), paclitaxel (Taxol; Sigma Aldrich, USA) and bleomycin (BLM; Huayao Zhushi Association, Japan) at varying plasma peak concentrations (PPC) of 0.01 PPC, 0.1 PPC, 1.0 PPC, 10.0 PPC, and the cells were incubated for another 48 h. Afterwards, the cells were stained with 20 μl of 5.0 mg/ml sterile MTT solution (3-[4,5-dimethylthiazol-2-yl]-2,5-diphenyltetrazolium bromide; Sigma) for 4 h at 37°C, after which the medium was removed and thoroughly mixed with 100 μl dimethyl sulfoxide (DMSO) to dissolve formazan crystals. The cells were then agitated for 10 min, and their absorbance was measured at 490 nm using a spectrophotometric microplate reader (Bio-Rad Inc., USA). Each treatment group was analyzed in triplicate, and the experiment was repeated 3 times. The inhibition ratio for the tumor cells at each drug concentration was calculated using the following formula: inhibition ratio (%) = (1- average OD value of the experimental cells/average OD value of the control cells) × 100. The half maximal inhibitory concentration (IC_50_) of each chemotherapeutic drug was determined from the dose-response curve constructed according to the inhibition ratio for each concentration. The resistance index (RI) for cells was calculated using the following formula: RI = IC_50 _of the experimental cells/IC_50 _of the control cells.

### Statistical analysis

Statistical analysis was conducted using SPSS 16.0 software. The results are presented as the mean ± standard deviation. The ANOVA and the Student's *t*-test were used to compare mean values between groups. Two-sided probability values of less than 0.05 were considered statistically significant.

## Results

### Production of recombinant adenoviruses in HEK 293 cells

**Figure 1:**
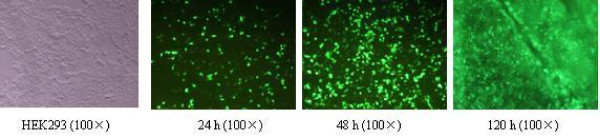
**GFP expression in HEK 293 cells transducted with the recombinant adenoviruses Ad-GFP-HA117, Ad-GFP-MDR1 or Ad-GFP (×100)**.

The recombinant adenoviruses Ad-GFP-HA117, Ad-GFP-MDR1, and Ad-GFP were transducted into HEK 293 cells. After 4-5 d, the transducted cells could be observed floating in the media under a fluorescence microscope (Figure. 1). The viral titers of Ad-GFP-HA117, Ad-GFP-MDR1 and Ad-GFP ranged between 2.5-3.5 × 10^9 ^plaque forming units (PFU)/ml.

### Fluorescence and adenovirus quantification in 4T1 cells

**Figure 2:**
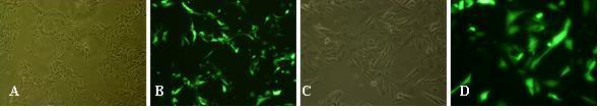
**GFP expression in 4T1 cells 48 h after transduction**. A: 4T1 cells (×100); B: 4T1/HA117, 4T1/MDR1 or 4T1/GFP transductants (×100); C: 4T1 cells (×200); D: 4T1/HA117, 4T1/MDR1 or 4T1/GFP transductants (×200). We show only one figure of the all three transductants' microscope images because of the limination of length.

**Figure 3:**
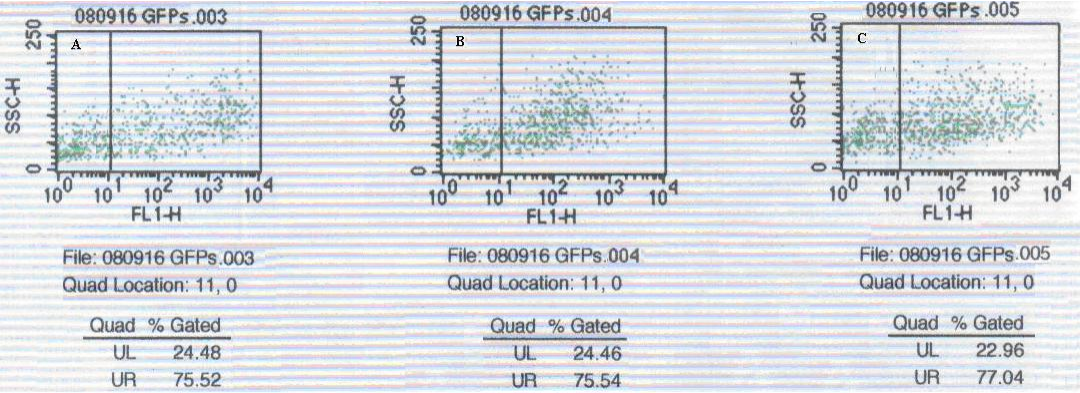
**Transduction efficiency of 4T1 cells 48 h after transduction with Ad-GFP-HA117, Ad-GFP-MDR1 or Ad-GFP at a MOI = 50**. A: Transduction efficiency of Ad-GFP-HA117 in 4T1/HA117 cells; B: Transduction efficiency of Ad-GFP-MDR1 in 4T1/MDR1 cells; C: Transduction efficiency of Ad-GFP in 4T1/GFP cells. The number of cells is shown on the × axis. UR and UL indicate the cells with and without green fluorescence, respectively. Cells expressing GFP represent those that were successfully transducted. This experiment was repeated at least 3 times with the same results.

The expression of GFP in 4T1 cells was observed 48 h after transduction using a fluorescence microscope (Figure. 2). As shown in Figure 3, the transduction efficiencies of individual stable transductants were between 75- 80% when the adenovirus MOI = 50. In addition, the transduction efficiency increased with increasing concentration of adenovirus. Both the survival rate (over 80%) and the transduction efficiency (80%) of 4T1 cells were relatively high when the adenovirus MOI = 50. Thus, an MOI = 50 was used in further experiments.

### Up-regulation of HA117 and MDR1 mRNA and P-gp protein expression in 4T1 cells

**Figure 4:**
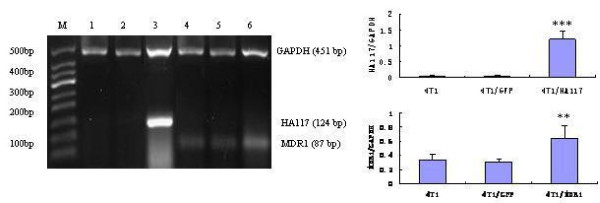
**The mRNA expression levels of the HA117 and MDR1 genes in 4T1 cells 48 h after transduction of Ad-GFP-HA117 or Ad-GFP-MDR1 as quantified by RT-PCR**. The levels of HA117 and MDR1 mRNA increased significantly 48 h after transduction. The expression of GAPDH mRNA was also examined and served as a loading control. The bar graphs represent the quantification and comparison of the signal intensity of the mRNA bands on the gel. M: 50-bp DNA ladder; 1: 4T1; 2: 4T1/GFP transfectants; 3: 4T1/HA117 transfectants; 4: 4T1 cells; 5: 4T1/GFP transfectants; 6: 4T1/MDR1 transfectants. P < 0.05 ** vs. control cells, P < 0.01*** vs. control cells. This experiment was repeated at least 3 times with the same results.

**Figure 5:**
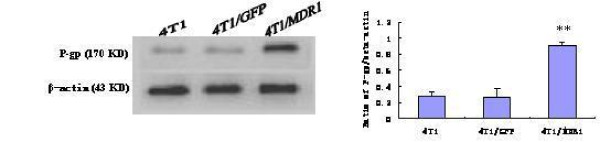
**The expression of P-gp as assessed by western blot analysis**. The levels of β-actin protein were also examined and served as a loading control. The expression of P-gp was upregulated in MDR1-transfected 4T1 cells. The bar graphs represent the quantification and comparison of the signal intensity of the bands on the immunoblots. P < 0.05** vs. control cells. This experiment was repeated at least 3 times with the same results.

To detect changes in the mRNA and protein levels of HA117 and MDR1 in 4T1 cells transducted with Ad-GFP-HA117, Ad-GFP-MDR1 or Ad-GFP viral supernatants for 48 h and RT-PCR and western blotting analysis were performed. However, we could not be detect because an antibody against this protein has not been synthesized. As shown in Figure 4, the mRNA levels of HA117 and MDR1 were remarkably higher in 4T1/HA117 and 4T1/MDR1 transductants than in 4T1 cells or 4T1/GFP transductants (P < 0.01 for HA117 and P < 0.05 for MDR1). In addition, western blotting analysis (Figure. 5) showed a corresponding increase change in P-gp expression in the 4T1/MDR1 transductants. Collectively, these results demonstrate that the expression of HA117 or MDR1 can be effectively up-regulated by recombinant adenovirus-mediated transduction of vectors expressing the HA117 or MDR1 genes, respectively.

### The HA117 gene has no drug-excretion function

**Figure 6:**
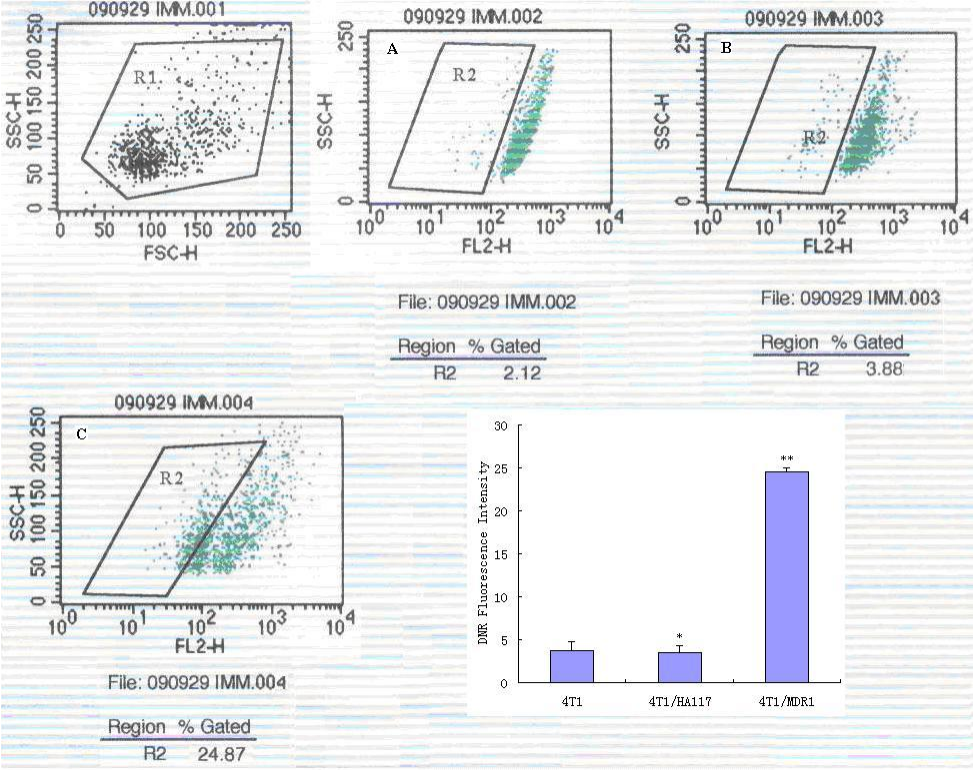
**Drug-elimination activity of HA117 and MDR1 as analyzed using the DNR efflux assay**. The fluorescence intensity of DNR in 4T1/MDR1 cells (C) was much lower than that of 4T1 (A) and 4T1/HA117 (B) cells (P < 0.05). There was no statistically significant difference in the DNR fluorescence intensity between 4T1 and 4T1/HA117 cells (P > 0.05). The bar graphs represent the quantification and comparison of the fluorescence intensity of the cells. P > 0.05* vs. control cells (4T1), P < 0.05 ** vs. control cells (4T1). R1: Percent of all cells. R2: Percent of cells with no or low DNR fluorescence. This experiment was repeated at least 3 times with the same results.

To explore the multidrug resistance mechanism of HA117 and assess whether its drug-induced activity is the same as that of MDR1, a DNR efflux assay was carried out to detect the DNR fluorescence intensity when 4T1 cells were transducted with the recombinant adenoviruses. As shown in Figure 6, there was no significant difference in the DNR fluorescence intensity between 4T1/HA117 and 4T1 cells (P > 0.05), whereas the difference between 4T1/MDR1 and 4T1 cells was significant (P < 0.05).

### Sensitivity to anticancer drugs

The MTT assay allowed us to determine the drug sensitivities of 4T1/HA117, 4T1/MDR1, 4T1/GFP and 4T1 cells to anticancer drugs **- **ADM, VCR, Taxol and BLM, which are the commonly used drugs in the therapy of breast cancer, especially the first three. On the other hand, ADM, VCR and Taxol are the substrates of P-gp and BLM is a P-gp non-substrate drug, which make them suitable to be investigated in our present study so as to evaluate the MDR function of HA117 comparing with that of MDR1. As shown in Table 1, both the HA117 and MDR1 transductants exhibited decreased sensitivity to the P-gp substrate drugs ADM, VCR and Taxol (P < 0.05). Interestingly, overexpression of HA117 also decreased the sensitivity of the transductants to the P-gp non-substrate drug BLM (P < 0.05). The IC_50 _and the RI of 4T1/HA117 cells was lower than that of 4T1/MDR1 cells for P-gp substrate drugs, but these differences were not statistically significant (P > 0.05). In contrast, the IC_50 _and the RI of 4T1/HA117 cells was higher than that of 4T1/MDR1 cells for P-gp non-substrate drugs and the difference was statistically significant (P < 0.05). This result supported our earlier finding that 4T1/HA117 and 4T1/MDR1 cells exhibit increased resistance to anticancer drugs.

**Table 1:** IC_50 _(μg/ml) for ADM, VCR, Taxol and BLM in 4T1, 4T1/HA117, 4T1/MDR1 and 4T1/GFP cells.

	ADM		VCR		Taxol		BLM	
	
Cell lines	IC_50 _(μg/ml)	**R.I**.	IC_50 _(μg/ml)	**R.I**.	IC_50 _(μg/ml)	**R.I**.	IC_50 _(μg/ml)	**R.I**.
4T1	0.4159 ± 0.0791	1	0.4775 ± 0.0757	1	0.0294 ± 0.0058	1	0.4789 ± 0.1104	1
4T1/HA117	**8.2369 ± 1.9458	19.8050	**4.3292 ± 0.4452	9.0663	**0.2859 ± 0.0479	9.7245	*1.7073 ± 0.4062	3.5650
4T1/MDR1	**10.0746 ± 1.0684	24.2236	**5.2754 ± 1.0974	11.0480	**0.3050 ± 0.1067	10.3741	0.4612 ± 0.0733	0.9630
4T1/GFP	0.5126 ± 0.1547	1.2325	0.4508 ± 0.1193	0.9441	0.0292 ± 0.0016	0.9932	0.4924 ± 0.1172	1.0282

Values are shown as the mean ± SD.

ADM: Adriamycin; VCR: vincristine; Taxol: paclitaxel; BLM: bleomycin. * P < 0.05 and ** P < 0.01 compared to the respective control group.

## Discussion

MDR is a phenomenon whereby tumor cells exposed to one cytotoxic agent develop cross-resistance to a range of structurally and functionally unrelated compounds. The exact mechanism of MDR in cancer cells is still under investigation, but many MDR-associated genes have been identified, as mentioned earlier [2-7]. The MDR of breast cancer cells to cytotoxic drugs has been linked to the over-expression of cell-surface P-gp, with more than 40% of breast cancers over-expressing P-gp [12]. P-gp is a member of the adenosine triphosphate (ATP)-dependent transporters that are known to confer cross-resistance to a variety of structurally unrelated cytotoxic drugs, such as anthracycline, taxanes, vinca alkaloids and other drugs widely used for cancer treatment [13-15]. Based upon these findings, we chose to investigate the effects of P-gp substrate (ADM, VCR and Taxol) and P-gp non-substrate (BLM) drugs on the survival of MDR1 and HA117 transducted cells.

ATRA, been a differentiation-inducing chemotherapeutic agent, is widely used for the treatment of acute promyelocytic leukemia (APL) and acute myeloid leukemia (AML), and often induces complete remission in most APL and AML patients [16-18]. However, clinical experience has shown that patients treated with ATRA alone does not remain on long-term remission and can in fact develop ATRA-resistant APL or AML [19]. The exact mechanism of ATRA resistance is still unknown, although many researchers have reported that resistance is caused by a point mutation in the PML/RARα fusion gene or by up-regulation of meningioma-1 gene (MN1) [20-22]. To elucidate the mechanism underlying ATRA resistance, we examined the gene library of ATRA-resistant HL-60 cells by suppressive subtractive hybridization and discovered the novel gene HA117, which is associated with ATRA resistance in HL-60 cells and MDR in both HL-60 and K562 cells. In the present study, we transducted recombinant adenoviral vectors encoding HA117 or MDR1 into breast cancer cell line 4T1 to investigate the MDR mechanism of HA117 and to perform a comparative study between HA117 and MDR1 in a solid tumor cell line.

Here, we transducted adenoviral vectors containing the GFP and HA117 genes or the GFP and MDR1 genes into 4T1 cells to generate the transductants 4T1/HA117 and 4T1/MDR1. The transduction efficiency and MOI were analyzed by fluorescence microscope and flow cytometry. Our results showed that the efficiency of transduction in 4T1 cells increased with increased concentration of the adenovirus; however, the number of dead cells increased when the MOI exceeded 50. Therefore, an MOI = 50 was chosen for further experiments. We found that transduction of 4T1 cells with HA117 or MDR1 significantly increased the transcription levels of both genes. We also evaluated the sensitivity of stable transductants to P-gp substrate (ADM, VCR, Taxol) and non-substrate (BLM) drugs. The results of the MTT assay revealed that MDR to P-gp substrate drugs was significantly enhanced in HA117- and MDR1-expressing cells when compared to their respective controls. There were no statistically significant differences in the IC_50 _or the RI of ADM, VCR, and Taxol between 4T1/HA117 and 4T1/MDR1 cells (P > 0.05), which indicates that the multidrug resistance strength of HA117 is similar to that of MDR1. It is clear that HA117 is a strong multidrug resistant novel gene and much importance should be given to it. In addition, the chemo-sensitivity of MDR1 transductants to the P-gp non-substrate drug BLM remained unchanged but decreased in HA117 transductants. This result is consistent with the results of the DNR efflux assay which demonstrated that the differences in the DNR fluorescence intensity between 4T1/HA117 and 4T1 cells were not statistically significant (P > 0.05), whereas the differences between 4T1/MDR1 and 4T1 cells were significantly significant (P < 0.05). These results suggest that HA117 has no drug-excretion function and that it may not generate MDR in breast cancer cells using the same mechanism as MDR1. So far, the specific mechanism by which HA117 promotes MDR is still unclear. Therefore, additional studies are required to determine the exact mechanism of MDR of HA117 including its association with the prognosis of AML and whether it can promote drug resistance in tumor cells in vivo.

## Conclusions

Our study confirms that transduction of HA117- or MDR1-expressing recombinant adenoviruses into breast cancer cells can increase the transcription of these genes and confer the breast cancer cells drug resistance. Moreover, the drug resistance of HA117 is similar to that of MDR1, which makes it clear that HA117 is a strong multidrug resistance related novel gene. Our results also show that HA117-induced MDR does not involve an increase in the efflux of cytotoxic compounds out of the cells. Overall, our study provides further insights into the nature of HA117 and highlights the importance of choosing the appropriate chemotherapeutic drugs for the treatment of cancer patients.

## Competing interests

The authors declare that they have no competing interests.

## Authors' contributions

LHZ designed and conducted the experiments, acquisited and analyzed the data and drafted the paper; XQJ and YHX designed and developed the concept of this work and gave final approval; YXG, RL, ZHG, TFC, YHS and XHD assisted in acquisition, analysis and interpretation of data and revised and polished the report. All authors have seen and approved the final manuscript.

## Disclosure statement

We promise that the article is original, is not under consideration, or has not been published previously elsewhere, and its content has not been anticipated by a previous publication. There are no benefits conflicts in any form.
